# Characteristics and Survival Outcomes of Hepatocellular Carcinoma After the Fontan Operation

**DOI:** 10.1016/j.jacadv.2025.101646

**Published:** 2025-03-12

**Authors:** Benjamin E. Rosenthal, Maarouf A. Hoteit, Gentian Lluri, Christiane Haeffele, Tami Daugherty, Richard A. Krasuski, John D. Serfas, R. Andrew de Freitas, Avaliese Porlier, Adam M. Lubert, Fred M. Wu, Anne Marie Valente, Eric V. Krieger, Yonatan Buber, Fred H. Rodriguez, Scott Gaignard, Anita Saraf, Morgan Hindes, Michael G. Earing, Matthew J. Lewis, Marlon S. Rosenbaum, Ali N. Zaidi, Kali Hopkins, Elisa A. Bradley, Ari M. Cedars, Jong L. Ko, Wayne J. Franklin, Abby Frederickson, Salil Ginde, Jasmine Grewal, Annique Nyman, Jungwon Min, Charlotte Schluger, Elizabeth Rand, Moira Hilscher, Jack Rychik, Yuli Y. Kim

**Affiliations:** a Department of Medicine, Hospital of the University of Pennsylvania, Philadelphia, Pennsylvania, USA; b Division of Hepatology, Hospital of the University of Pennsylvania, Philadelphia, Pennsylvania, USA; c Ahmanson/UCLA Adult Congenital Heart Disease Center, Division of Cardiology, Department of Medicine, University of California Los Angeles, Los Angeles, California, USA; d Division of Cardiovascular Medicine, Stanford Medicine, Stanford, California, USA; e Duke University Health System, Durham, North Carolina, USA; f Division of Pediatric Cardiology, Anne & Robert H. Lurie Children's Hospital of Chicago, Chicago, Illinois, USA; g Department of Pediatrics, Cincinnati Children's Hospital Heart Institute, University of Cincinnati, Cincinnati, Ohio, USA; h Division of Cardiology, Department of Medicine, Brigham and Women's Hospital, Boston, Massachusetts, USA; i Department of Cardiology, Boston Children's Hospital, Boston, Massachusetts, USA; j University of Washington Medical Center, University of Washington School of Medicine, Seattle, Washington, USA; k Seattle Children's Hospital, Seattle, Washington, USA; l Department of Pediatrics, Emory University, Atlanta, Georgia, USA; m Department of Pediatrics, University of Pittsburgh Medical Center, Pittsburgh, Pennsylvania, USA; n The University of Chicago Medicine, Chicago, Illinois, USA; o Division of Cardiology, Columbia University Irving Medical Center, New York, New York, USA; p Mount Sinai Adult Congenital Heart Disease Center, Mount Sinai Heart, New York, New York, USA; q Division of Cardiovascular Medicine, The Ohio State University, Columbus, Ohio, USA; r Department of Medicine, Johns Hopkins Hospital, Baltimore, Maryland, USA; s Department of Pediatrics, Johns Hopkins Hospital, Baltimore, Maryland, USA; t Division of Pediatric Cardiology, Phoenix Children's Hospital, Phoenix, Arizona, USA; u Department of Child Health, University of Arizona College of Medicine-Phoenix, Phoenix, Arizona, USA; v Department of Pediatrics, Children's Hospital of Wisconsin, Milwaukee, Wisconsin, USA; w Division of Cardiology, St. Paul's Hospital, University of British Columbia, Vancouver, British Columbia, Canada; x Division of Cardiology, Children's Hospital of Philadelphia, Philadelphia, Pennsylvania, USA; y Department of Biomedical and Health Informatics, Children's Hospital of Philadelphia, Philadelphia, Pennsylvania, USA; z Division of Hepatology, Children's Hospital of Philadelphia, Philadelphia, Pennsylvania, USA; aa Division of Cardiology, Hospital of the University of Pennsylvania, Philadelphia, Pennsylvania, USA

**Keywords:** congenital heart disease, Fontan-associated liver disease, Fontan operation, hepatocellular carcinoma, single ventricle

## Abstract

**Background:**

The Fontan operation is a surgical procedure to palliate single ventricle congenital heart disease. Hepatocellular carcinoma (HCC) is a rare complication of Fontan-associated liver disease (FALD).

**Objectives:**

The authors aim to examine characteristics of individuals with Fontan circulation diagnosed with HCC and to describe tumor characteristics, treatment, and survival outcomes of these patients.

**Methods:**

This was a multicenter retrospective case-control study of adults with Fontan circulation between 2005 and 2021. HCC cases were included based on histology or imaging-based diagnosis. Controls were randomly selected in a 3:1 ratio from the center in which the case was derived. Descriptive statistics were used to compare groups and Kaplan-Meier survival analysis was performed.

**Results:**

There were 58 cases of HCC diagnosed at a median age of 31 (IQR: 26-38) years. Diagnosis was made at very early or early stage disease in 68%. Compared to controls, cases had higher prevalence of advanced FALD including varices, ascites, splenomegaly, and decreased platelets. Treatment with curative intent (combined heart-liver transplantation, resection, or ablation) was performed in 41%. Survival at 1 year was 78.9% and highest among those diagnosed at very early or early stage. Over half were undergoing active surveillance at diagnosis, which showed a nonsignificant trend toward higher survival (*P* = 0.088).

**Conclusions:**

We describe the clinical characteristics, treatment, and survival in patients with FALD-HCC. Results suggest that adults with FALD-HCC diagnosed with early stage disease may have survival benefit. Our findings underscore the importance of HCC screening for early detection in individuals after the Fontan operation.

The Fontan operation is a palliative procedure to treat those born with complex congenital heart disease and results in single ventricle physiology that is injurious to the liver.^1^ Fontan-associated liver disease (FALD)^2^ is characterized by fibrosis and/or cirrhosis and can be complicated by hepatocellular carcinoma (HCC).^3^ The first report of HCC in a patient with Fontan circulation (FC) was documented in 2005 and described a 23-year-old who died of hemoperitoneum from a ruptured tumor found on autopsy.^4^ Since then, a number of case reports have been published which describe a variety of clinical presentations, diagnostic approaches, treatments strategies, and outcomes. Data from a recent study from our consortium suggest that HCC is more likely to develop in patients with advanced FALD (cirrhosis by imaging and biopsy, evidence of portal hypertension including varices, ascites, splenomegaly, and thrombocytopenia), lower oxygen saturations, “older style” Fontan connections such as atriopulmonary Fontan, and history of prior Fontan revision or valve surgery^5^ but risk factors for carcinogenesis and natural history of disease remain incompletely understood. Incidence of HCC in the Fontan population is estimated to be 1.5% to 5% per year, and reported survival after diagnosis with HCC is poor.^5^, ^6^, ^7^, ^8^, ^9^, ^10^ A 2018 case series of 33 individuals with FC demonstrated a 1-year survival of 53% which was notably worse in those with symptoms of liver dysfunction.^11^

Given these knowledge gaps, the objectives of this study were to: 1) characterize adults with FC who are diagnosed with HCC; and 2) describe diagnosis, treatment, and survival outcomes of these patients.

## Methods

### Study design and data source

This was a multicenter, retrospective case-control study of patients ≥18 years old with FC followed in participating centers of the Alliance for Adult Research in Congenital Cardiology between 2005 and 2021. HCC cases were identified by either histology (biopsy, hepatectomy, liver explant, or autopsy) or imaging. Imaging diagnosis was defined as focal liver lesion(s) by multiphase computed tomography (CT) or magnetic resonance imaging (MRI) that fulfill standard Liver Reporting and Data System (LI-RADS)-5 criteria,^12^ plus one of the following: alpha-fetoprotein (AFP) >20 ng/mL or evidence of lesion growth ≥50% in a period of 6 months or less or growth ≥100% in a period of over 6 months on serial imaging studies.^12^ HCC cases meeting both histology and imaging criteria were categorized as histologic diagnosis for analysis. Laboratory, pathology, and radiology reports for all HCC cases were reviewed and confirmed by a board-certified hepatologist with expertise in HCC and FALD (M.H.).

Controls were randomly selected in a 3:1 ratio from each center including all patients ≥18 years old with FC followed at the site from which the case was derived. Inclusion criteria were abdominal imaging (ultrasound, CT, or MRI) without a focal liver lesion (or if present, imaging evidence of stable size over at least 24 months) and blood work within 18 months of the last clinic visit. Those without imaging or labs, AFP ≥10 ng/dL at any time, or history of hepatitis B or C were excluded.

Each center abstracted demographic and clinical characteristics from the electronic health record and data were entered into a secure online database using Research Electronic Data Capture tools.^13^ Children's Hospital of Philadelphia served as the coordinating site and Hospital of the University of Pennsylvania as the collaborating site. The study was approved by the Institutional Review Board at Children's Hospital of Philadelphia and by respective research oversight boards at the participating centers and collaborating site with waived consent.

### Clinical variables

Data collected included patient demographics, surgical history, primary cardiac diagnosis, hepatic comorbidities, and cardiac comorbidities. Laboratory, cardiac catheterization, echocardiogram, and cardiopulmonary exercise stress tests within 18 months of HCC diagnosis prior to treatment (cases) or within 18 months of last outpatient visit (controls) were abstracted. Aspartate transaminase (AST) to platelet ratio index (APRI)^14^ and fibrosis-4 index (FIB-4)^15^ scores and Model for End-stage Liver Disease eXcluding INR (MELD-XI)^16^ were calculated as previously described. APRI, FIB-4, and MELD-XI were collapsed into binary (low/high) variables using validated cutoffs of <0.5 and ≥0.5, <1.45 and ≥1.45, and <11 and ≥11, respectively.^17^, ^18^, ^19^ The VAST (Varices, Ascites, Splenomegaly, Thrombocytopenia) score, which assesses signs of portal hypertension, was calculated for each patient and “advanced FALD” was defined as VAST score ≥2.^20^

Ultrasound, MRI, and CT radiology reports were reviewed to determine presence of cirrhotic morphology, portosystemic collateral circulation, and spleen size in largest dimension. Any radiographic evidence of esophageal, gastric, splenic, or other varices were coded as having portosystemic shunting, regardless of size or clinical significance. Splenomegaly was defined as largest diameter >12 cm. Liver biopsy results were reviewed and degree of fibrosis was categorized as none, portal/central, septal, bridging, or cirrhosis.^21^

### HCC variables

Variables collected at HCC diagnosis included mode of diagnosis, number of tumor lesions, size of tumor lesions, Child-Turcotte-Pugh (CTP) scores and categories, and Barcelona Clinic Liver Cancer (BCLC) stage.^22^ BCLC is a comprehensive, validated staging system for liver cancer predictive of survival that includes tumor characteristics (eg, tumor size, vascular invasion, extrahepatic spread), patient's liver function (CTP score), and functional capacity. Patients were defined as being under “active surveillance” for HCC if they underwent screening ultrasound or other cross-sectional abdominal imaging (CT or MRI) 3 to 12 months prior to diagnosis, consistent with current guidelines for patients at higher risk for developing HCC.^23^ HCC treatment delivered included surgical therapy (liver transplant or resection), liver-directed therapy (LDT) (percutaneous ablation, transarterial chemoembolization [TACE], transarterial radioembolization, or external radiotherapy), systemic therapy (chemotherapy or immunotherapy), and symptomatic/palliative therapy. Those who underwent multiple treatment modalities were assigned the most definitive therapy as follows: liver transplant, surgical resection, ablation, other LDT, systemic therapy, and symptomatic/palliative therapy. Transplant, resection, and percutaneous ablation were considered to be treatment with curative intent.^24^ Survival was captured by alive or deceased status at the last follow-up.

### Statistical analysis

Clinical characteristics were described as frequency and percent for categorical variables. Continuous variables were summarized as median (IQR). We examined the association of demographic, clinical, and imaging characteristics between cases and controls using the Wilcoxon rank sum tests, Pearson's chi-squared tests, and Fisher's exact tests. Univariate logistic regression was used to assess the association between active surveillance and being diagnosed at very early or early stage HCC using ORs and 95% CI.

The primary outcome of interest was survival after HCC diagnosis and the exposure was BCLC stage. Kaplan-Meier curves and log-rank tests were used to compare crude survival rates. Up to 2 potential confounders were chosen based on clinical relevance and examining differences in baseline demographic and cardiac comorbidities. A Cox proportional hazards regression model was used to evaluate the adjusted association between BCLC stage and survival. All covariates were assessed for proportional hazards assumptions. A secondary exploratory analysis of crude survival stratified by treatment and surveillance status was performed using Kaplan-Meier curves and log-rank tests.

We conducted statistical analyses using R 4.1.3 (R Core Team, 2020) and Stata v17 (StataCorp LLC, 2021).

## Results

### Clinical characteristics

There were 58 HCC cases in adults with FC from 18 North American centers. The number of HCC cases submitted from each center ranged from 1 to 10. HCC cases were more prevalent in the latter years with 7% diagnosed in first half of the study period (2005-2012) and 93% in the second half (2013-2021).

Clinical characteristics are described in Table 1. The median age at HCC diagnosis in the cases (43% female and 79% White) was 31 (IQR: 26-38) years. There was no significant difference between cases and controls regarding age, time since Fontan, age at Fontan, race, or primary cardiac diagnosis. Compared to controls, cases more frequently had undergone right atrium to pulmonary artery or right atrium to right ventricle Fontan repair. Prior Fontan revision was more prevalent (36% vs 17%, *P* = 0.003). Cases also had a higher prevalence of cardiac comorbidities including arrhythmias (69% vs 48%, *P* = 0.006), desaturation (32% vs 16%, *P* = 0.009), and heart failure (24% vs 8%, *P* < 0.001), despite comparable echocardiographic and hemodynamic parameters by cardiac catheterization.

**Table 1:** Patient Characteristics and Testing of HCC Cases and Controls (n = 232)

	Median (IQR) or n (%)			*P* Value
	Total (N = 232)	Controls (n = 174)	Cases (n = 58)	
Patient characteristics				
Age (y)	30 (24-38)	30 (24-38)	31 (26-38)	0.500
Female	111 (48)	86 (49)	25 (43)	0.400
Race (White)	187 (81)	141 (81)	46 (79)	0.800
Body mass index (kg/m^2^)	24.9 (22.0-28.3)	25.1 (22.1-29.0)	24.4 (21.8-27.0)	0.200
Age at Fontan (mo)	44 (27-89)	44 (29-90)	42 (26-81)	0.600
Time since Fontan (y)	26 (21-32)	25 (20-31)	27 (22-32)	0.120
Primary cardiac diagnosis				>0.90
Tricuspid atresia	70 (30)	50 (29)	20 (34)	
Double inlet left ventricle	46 (20)	34 (20)	12 (21)	
Hypoplastic left heart syndrome	37 (16)	26 (15)	11 (19)	
Double outlet right ventricle	25 (11)	20 (11)	5 (9)	
PA/IVS	17 (7)	13 (8)	4 (7)	
Unbalanced atrioventricular canal	16 (7)	11 (7)	5 (9)	
Hypoplastic tricuspid valve/RV	9 (4)	8 (5)	1 (2)	
Complex 2 ventricle (ie, superior-inferior ventricles)	5 (2)	5 (3)	0 (0)	
Hypoplastic mitral valve/LV	3 (1)	3 (2)	0 (0)	
Mitral atresia	1 (0.4)	1 (1)	0 (0)	
Other	3 (1)	3 (2)	0 (0)	
Heterotaxy	20 (9)	15 (9)	5 (9)	>0.90
Fontan type				**0.027**
Lateral tunnel	94 (41)	75 (43)	19 (33)	
Extracardiac	65 (28)	52 (30)	13 (22)	
Right atrium-pulmonary artery	62 (27)	40 (23)	22 (38)	
Right atrium-right ventricle	4 (12)	1 (1)	3 (5)	
Other	7 (3)	6 (3)	1 (2)	
Warfarin or direct oral anticoagulant	95 (41)	72 (41)	23 (40)	0.800
Cardiac comorbidities				
Arrhythmias	124 (53)	84 (48)	40 (69)	**0.006**
Fontan catheter intervention	109 (47)	77 (44)	32 (55)	0.150
Patent fenestration	83 (36)	54 (31)	29 (50)	**0.009**
Pacemaker	74 (32)	57 (33)	17 (29)	0.600
Desaturation (saturation <90%)	46 (20)	28 (16)	18 (32)	**0.009**
Fontan revision	51 (22)	30 (17)	21 (36)	**0.003**
History of thrombus	24 (10)	19 (11)	5 (9)	0.600
Transient ischemic attack or stroke	28 (12)	22 (13)	6 (10)	0.600
Heart failure	27 (12)	13 (8)	14 (24)	**<0.001**
PLE or plastic bronchitis	17 (7)	8 (5)	9 (16)	**0.016**
Valve surgery	15 (7)	7 (4)	8 (14)	**0.014**
Hepatic comorbidities				
Ascites	40 (17)	21 (12)	19 (33)	**<0.001**
Varices	37 (16)	19 (11)	18 (31)	**<0.001**
Gastrointestinal bleed	5 (2)	3 (2)	2 (3)	0.600
Hepatic encephalopathy	5 (2)	2 (1)	3 (5)	0.100
Therapeutic paracentesis	4 (2)	3 (2)	1 (2)	>0.90
Alcohol abuse	4 (2)	3 (2)	1 (2)	>0.90
Hepatitis C	3 (1)	0 (0)	3 (5)	**0.015**
Diabetes	2 (1)	2 (1)	0 (0)	>0.90
Spontaneous bacterial peritonitis	2 (1)	1 (1)	1 (1)	0.400
Labs				
Total bilirubin (mg/dL)	1.00 (0.70, 1.50)	0.90 (0.70, 1.40)	1.22 (0.80, 1.73)	**0.010**
AST (IU/L)	26 (22, 32)	25 (21, 32)	29 (23, 36)	**0.010**
ALT (IU/L)	28 (21, 36)	27 (21, 36)	29 (22, 36)	0.600
Alkaline phosphatase (IU/L)	84 (64, 106)	84 (63, 103)	92 (73, 116)	**0.028**
Albumin (g/dL)	4.50 (4.20, 4.80)	4.60 (4.30, 4.90)	4.40 (3.90, 4.80)	**0.021**
Platelets (10^3^/μL)	165 (123, 212)	172 (135, 216)	136 (96, 194)	**<0.001**
AFP (ng/mL)	3 (2, 6)	3 (2, 4)	77 (8, 1,191)	**<0.001**
APRI				**<0.001**
<0.5	154 (68)	129 (75)	25 (45)	
≥0.5	74 (32)	44 (25)	30 (55)	
FIB-4				**<0.001**
<1.45	171 (75)	143 (83)	28 (52)	
≥1.45	56 (25)	30 (17)	26 (48)	
MELD-XI				**0.004**
<11	144 (63)	118 (69)	26 (47)	
≥11	83 (37)	54 (31)	29 (53)	
Abdominal imaging (US, CT, or MRI)				
Cirrhosis	150 (65)	99 (57)	51 (91)	**<0.001**
Splenomegaly	57 (25)	29 (17)	28 (48)	**<0.001**
Portosystemic shunting	39 (17)	15 (9)	24 (41)	**<0.001**
VAST score ≥2	59 (25)	30 (17)	29 (50)	**<0.001**
Liver biopsy (n = 77)				**0.013**
No fibrosis	5 (6)	3 (7)	2 (7)	
Portal/septal fibrosis	15 (19)	13 (28)	2 (7)	
Bridging fibrosis	34 (44)	22 (48)	12 (39)	
Cirrhosis	23 (30)	8 (17)	15 (48)	
Echocardiography				
Systemic ventricular dysfunction ≥ mild	67 (29)	53 (30)	14 (24)	0.400
AV valve regurgitation ≥ moderate	33 (14)	24 (14)	9 (16)	0.700
Aortic/neoaortic regurgitation ≥ moderate	14 (6.0%)	11 (6.3%)	3 (5.2%)	>0.90
Cardiac catheterization (n = 166)				
Fontan pressure (mm Hg)	14.0 (11.0, 16.0)	13.0 (11.0, 16.0)	15.0 (12.0, 17.0)	0.091
Right pulmonary artery pressure (mm Hg)	13.0 (11.0, 15.0)	13.0 (11.0, 15.0)	14.0 (11.0, 17.0)	0.120
Left pulmonary artery pressure (mm Hg)	13.0 (11.0, 15.0)	13.0 (11.0, 15.0)	15.0 (11.0, 17.0)	0.058
Right PCWP (mm Hg)	9.0 (6.2, 12.0)	8.0 (6.0, 11.0)	10.0 (7.0, 12.5)	0.086
Left PCWP (mm Hg)	9.0 (7.0, 11.8)	9.0 (7.0, 11.0)	11.0 (7.0, 13.0)	0.110
Ventricular EDP (mm Hg)	9.0 (7.0, 12.0)	9.0 (7.0, 11.0)	11.0 (8.0, 13.5)	0.082
SVC saturation (%)	68.0 (64.0, 73.0)	68.0 (64.0, 73.0)	67.0 (62.0, 73.0)	0.600
Right pulmonary artery saturation (%)	70.0 (65.0, 73.0)	69.0 (65.0, 73.0)	70.0 (67.0, 72.0)	0.800
Left pulmonary artery saturation (%)	69.0 (66.0, 73.0)	69.0 (66.0, 73.0)	70.2 (67.2, 73.0)	0.300
Aortic saturation (%)	92.0 (90.0, 94.0)	92.0 (90.0, 95.0)	92.0 (89.0, 94.0)	0.400
PVR (Wood units)	1.08 (0.79, 1.40)	1.11 (0.80, 1.50)	1.00 (0.63, 1.28)	0.130
Cardiac Index (L/min/m^2^)	2.60 (2.20, 3.10)	2.60 (2.20, 3.10)	2.70 (2.24, 3.12)	0.600
Cardiopulmonary exercise stress test (n = 169)				
Peak oxygen consumption (mL/kg/min)	21 (18, 26)	22 (18, 27)	20 (18, 23)	0.130

Values are median (IQR) or n (%).

AFP = alpha-fetoprotein; ALT = alanine transaminase; APRI = AST to Platelet Ratio Index; AST = aspartate transaminase; AV = atrioventricular; CT = computed tomography; EDP = end-diastolic pressure; FIB-4 = fibrosis- 4; LV = left ventricle; MELD-XI = Model for End-stage Liver Disease eXcluding International Normalized Ratio; MRI = magnetic resonance imaging; PA/IVS = pulmonary atresia with intact ventricular septum; PCWP = pulmonary capillary wedge pressure; PLE = protein losing enteropathy; PVR = pulmonary vascular resistance; RV = right ventricle; SVC = superior vena cava; US = ultrasound; HCC = hepatocellular carcinoma.

At the time of diagnosis, 72% of HCC cases had CTP class A (compensated) liver disease. The remainder, 28%, were class B (early decompensated) liver disease, and no patients met criteria for class C (late decompensated) liver disease. FIB-4 score ≥1.45 was found in greater proportion of cases compared to controls (48% vs 17%, *P* < 0.001) with a similar pattern for APRI ≥0.5 (55% cases vs 25% controls, *P* < 0.001) and MELD-XI score ≥11 (53% cases vs 31% controls, *P* = 0.004). Median AFP was 77 (IQR: 8-1,191) ng/mL in cases at diagnosis with 15 cases (29%) having AFP <10 ng/mL.

There was a higher prevalence of advanced FALD (VAST score ≥2) in HCC cases than in controls (50% vs 17%, *P* < 0.001). Compared to controls on cross-sectional imaging (MRI and/or CT), cases had more evidence of cirrhosis (91% vs 57%, *P* < 0.001), splenomegaly (48% vs 17%, *P* < 0.001), and portosystemic shunting (41% vs 9%, *P* < 0.001). On liver biopsy, cases were more likely to have cirrhosis (48% vs 17%) and less likely to have portal/septal fibrosis (7% vs 28%, *P* = 0.013 for both). Cases had significantly higher bilirubin, lower albumin, and platelet levels, all consistent with more advanced liver disease. Other comorbidities of cirrhosis such as encephalopathy, gastrointestinal bleeding, and spontaneous bacterial peritonitis, however, were rare overall.

Diagnosis and treatment data are summarized in Table 2. A diagnosis by histology was made in 36 (62%) cases and a diagnosis was made by imaging in 22 (38%). Most patients were diagnosed at very early (BCLC 0) or early (BCLC A) stages in 9 (16%) and 30 (52%), respectively. Of 38 patients who had cross-sectional imaging available at the time of diagnosis, 12 (32%) had 2 or more tumors demonstrated on imaging. Median tumor size was 3.9 (IQR: 2.2-5.4) cm. Vascular invasion was present in 10% and metastatic disease in 5%.

**Table 2:** HCC Diagnosis and Treatment (N = 58)

Mode of diagnosis (frequency)	
Imaging	34 (59)
Biopsy	26 (45)
Explant or hepatectomy	9 (16)
Autopsy	1 (2)
Mode of diagnosis^a^	
Histology	36 (62)
Imaging	22 (38)
Active HCC surveillance	31 (54)
BCLC stage at diagnosis	
0	9 (16)
A	30 (52)
B	8 (14)
C	6 (10)
D	3 (5)
Missing	2 (3)
Treatment (frequency)	
Surgery	
Resection	10 (17)
Liver transplant	9 (16)
Liver-directed therapy	
Transarterial chemoembolization	17 (29)
Percutaneous ablation	8 (14)
Transarterial radioembolization	7 (12)
External radiotherapy	6 (10)
Systemic therapy	14 (24)
Symptomatic/palliative	4 (67)
Treatment (most definitive)	
Liver transplant	9 (16)
Resection	9 (16)
Ablation	6 (10)
Other liver-directed therapy	22 (38)
Systemic therapy	3 (5)
Symptomatic/palliative^b^	9 (16)

Values are n (%).

BCLC = Barcelona Clinic Liver Cancer; other abbreviation as in Table 1.

a Cases diagnosed by both imaging and histology were grouped as histology.

b One patient was diagnosed at autopsy and was included in the symptomatic/palliative treatment group.

Of the 58 cases, 31 (54%) were undergoing active HCC surveillance at the time of diagnosis. This group had a higher prevalence of HCC diagnosed at very early or early stages (BCLC 0 or A) compared to those who were not under active surveillance (84% vs 52%, *P* = 0.010). The odds of being diagnosed at an earlier stage HCC was 4.8 times higher for individuals under active surveillance compared to those not under active surveillance (OR: 4.8; 95% CI: 1.39-16.5; *P* = 0.013). There was no association between active surveillance and either number of tumors at diagnosis (*P* = 0.463) or metastatic disease or tumor invading vasculature (*P* = 0.159).

### HCC treatment

Table 2 demonstrates that patients with FC and HCC underwent multiple treatment modalities. When considered by most definitive, surgical therapy was pursued in 18 (31%) cases, one-half of whom underwent transplant. All transplants were performed as combined heart-liver transplant (CHLT) operations. One patient underwent resection followed by transplant and was grouped under the latter category. Among the 9 patients who underwent CHLT, 3 had known HCC prior to referral for CHLT, with the remaining 6 diagnosed by pathologic examination of the explanted liver. Percutaneous ablation and nonablative LDT comprised definitive therapy in 6 (10%) and 22 (38%), respectively. The most common nonablation LDT was TACE. Only 3 (5%) received systemic therapy alone and 9 (16%) received symptomatic/palliative therapy.

**Figure 1:**
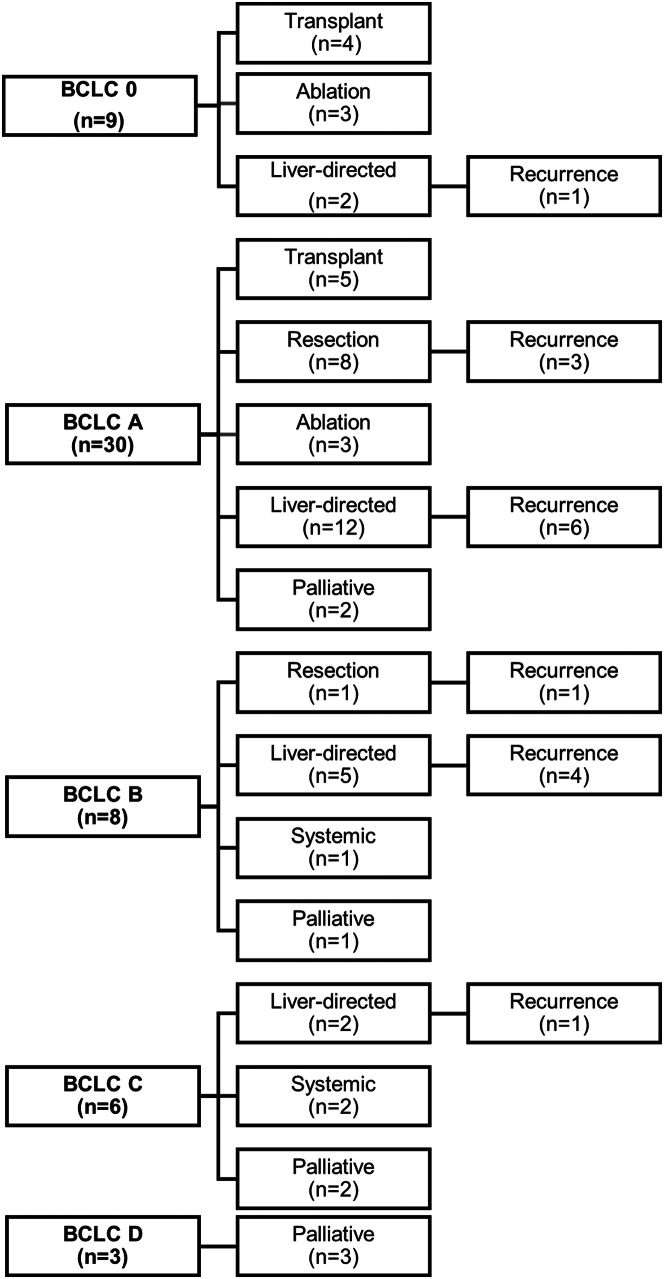
Flow Diagram for BCLC Staging, Treatment, and Recurrence at Last Follow-Up for the Cohort of Patients With HCC After Fontan Palliation BCLC staging was available for 56 patients.

Figure 1 shows treatment administered according to BCLC stage. Patients with very early or early stage diagnoses were more likely to receive therapy with curative intent. Of those diagnosed in BCLC stage 0 or A, 59% were treated with curative therapy compared to 13% in BCLC stage B and none in BCLC stage C (*P* < 0.001).

When analyzed by surveillance status, cases who had been under active surveillance received curative therapies more often than those who were not (58% vs 22%). Conversely, those not under active surveillance received more symptomatic/palliative therapy (26% vs 7%, *P* = 0.029). Patients under active surveillance had nearly 5 times higher odds of undergoing curative treatment compared to those who were not (OR: 4.85; 95% CI: 1.53-15.4; *P* = 0.007).

### HCC outcomes

Among the 9 cases who had resection as their most definitive treatment, there was recurrence of HCC in 4 (44%) but no recurrence in the 9 cases who underwent CHLT (Figure 1). Among the 22 who received nonablation LDT, 15 (68%) had evidence of viable tumor on the last available imaging, indicating an incomplete response to TACE, transarterial radioembolization, or radiotherapy.

Over a median follow-up of 17.3 (IQR: 6.5-34.9) months after HCC diagnosis, 21 out of 58 cases died, including one diagnosed at the time of autopsy, resulting in an overall survival of 63%. The 1- and 3-year survival rates were 78.9% and 63.7%, respectively. The incidence of death after HCC diagnosis was 155 cases per 1,000 person-years.

**Figure 2:**
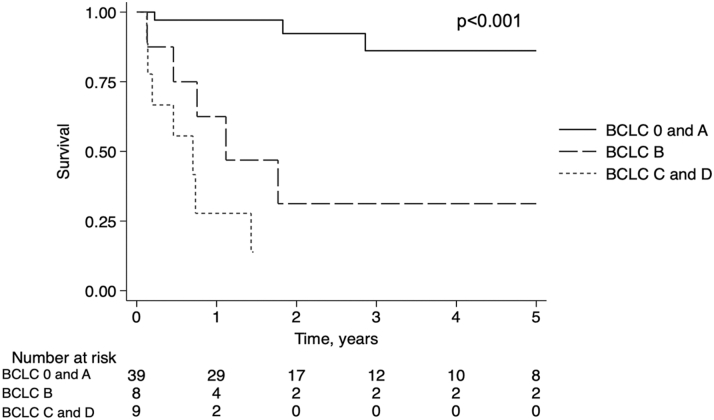
Kaplan-Meier Survival Analysis Grouped by BCLC Stage at Diagnosis Excluding Two Patients for Which There Were Missing Data (N = 56) Number at risk table and log-rank *P* value are shown.

When stratified by cancer stage at diagnosis, crude survival was highest in earlier BCLC stages and decreased in a stepwise fashion with each increase in disease stage. Cases with very early and early stage disease (BCLC 0 or A) had a median survival of 21.9 (IQR: 11.2-53.3) months with 1-year survival of 97%. Cases with intermediate stage (BCLC B) disease had a median survival of 11.3 (IQR: 7.3-41.9) months with 1-year survival of 62.5%. Those with advanced or end-stage disease (BCLC C or D, respectively) had a median survival of 6.5 (IQR: 2.3-8.8) months and 1-year survival of 27.8% (*P* < 0.001, Figure 2).

To adjust for potential confounders, we examined demographics and cardiac comorbidities of heart failure, desaturation, or pacemaker between those in BCLC stage 0 or A vs later stages but did not find significant differences (data not shown). In an unadjusted analysis, patients with intermediate stage HCC (BCLC stage B) had a five-fold increased risk of death (HR: 5.0; 95% CI: 1.6-15.9; *P* = 0.006) compared to those with BCLC 0 or A, while those with advanced or end-stage HCC (BCLC stage C or D) had a nearly 20-fold increased risk (HR: 19.7; 95% CI: 5.4-72.3; *P* < 0.001). After adjusting for age at diagnosis, the association remained significant for BCLC B (HR: 7.4; 95% CI: 2.1-26.9; *P* = 0.002) and BCLC C or D (HR: 19.8; 95% CI: 5.3-73.7; *P* < 0.001).

**Figure 3:**
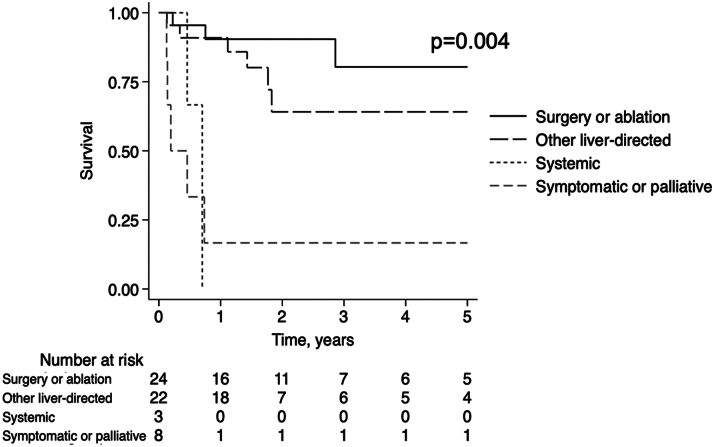
Kaplan-Meier Survival Analysis Excluding One Patient Diagnosed at Autopsy (n = 57) Stratified by Treatment: Curative Therapy (Resection, Liver Transplantation, and Ablation) vs Other Liver-Directed Therapy (TACE), TARE, External Radiotherapy), Systemic Therapy, and Symptomatic/Palliative Therapy Number at risk table and log-rank *P* value are shown.

**Figure 4:**
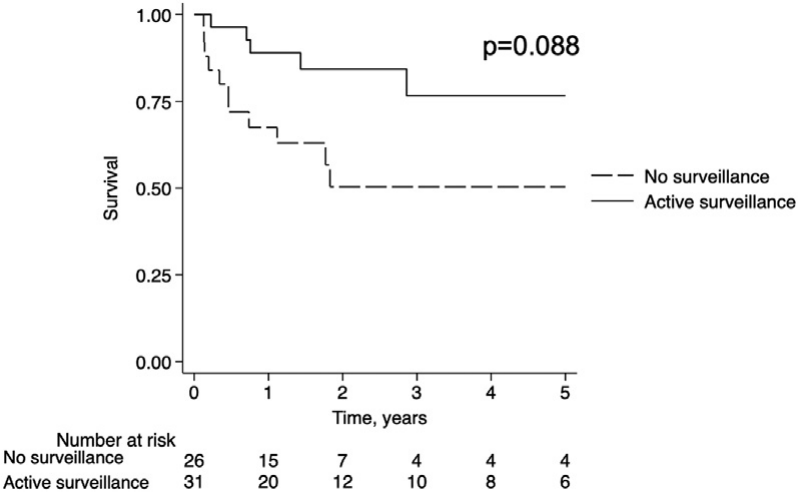
Kaplan-Meier Survival Analysis Excluding One Patient Diagnosed at Autopsy (N = 57) by Active HCC Surveillance Status Active surveillance was defined as ultrasound or cross-sectional imaging 3 to 12 months before diagnosis. Number at risk table and log rank *P* value are shown.

Over the entire study period, survival was 83% in those who received curative therapy (89% for transplant, 78% for resection, and 88% for ablation), compared to 59% in those who received nonablation LDT and 33% in those who after systemic and symptomatic/palliative therapy (*P* = 0.029). Though unadjusted 1-year survival was similar between curative therapy and other LDT (90.4% vs 90.9%, respectively), 3-year survival was higher in those who underwent curative therapy (80.4% vs 64%, *P* = 0.049). Figure 3 demonstrates significant differences in crude survival by treatment modality (*P* = 0.004). In another exploratory analysis, survival was examined by active surveillance status. Unadjusted survival was higher in those who had been under active surveillance prior to HCC diagnosis compared to those who were not, but did not reach significance (*P* = 0.088) (Figure 4).

## Discussion

We describe the clinical characteristics, diagnosis, and treatment outcomes of 58 adults with FC diagnosed with HCC across 18 North American centers between 2005 and 2021 (Central Illustration). Compared to controls, there was a high prevalence of atriopulmonary Fontan arrangements as well as prior Fontan revisions. Patients with HCC had higher burden of cardiac comorbidities and evidence of more advanced FALD. Those diagnosed with very early or early stage HCC appear to have higher survival compared to those diagnosed at later stages. As expected, patients who were diagnosed at early stages were more likely to have undergone curative therapy. Those who were under active surveillance were diagnosed at earlier stages and were more often treated with curative intent.

**Central Illustration:**
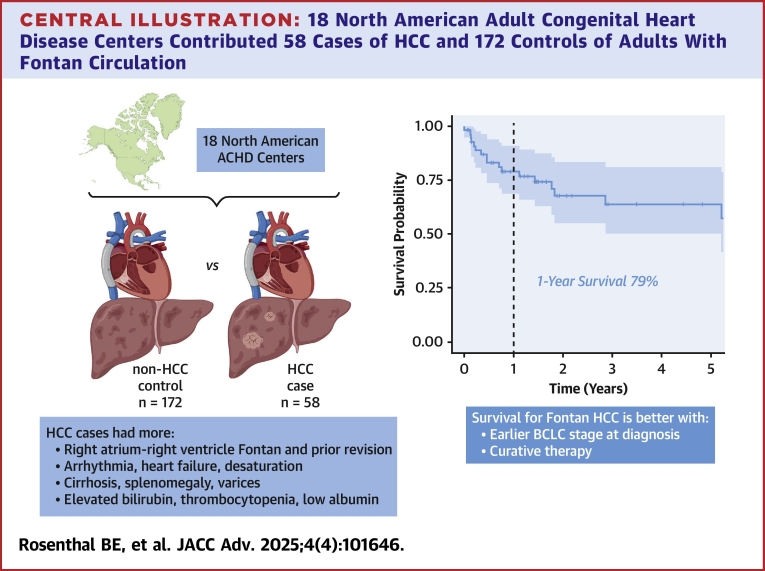
18 North American Adult Congenital Heart Disease Centers Contributed 58 Cases of HCC and 172 Controls of Adults With Fontan Circulation Clinical, laboratory, and imaging differences were found including higher prevalence of atriopulmonary Fontan and prior Fontan revision, greater burden of cardiac comorbidities, and evidence of worse FALD in HCC cases 1-year survival of Fontan HCC cases was 79%. ACHD = adult congenital heart disease; BCLC = Barcelona Clinic Liver Cancer Stage.

Prior to this study, the largest case series of HCC in Fontan patients was a multicenter study published by Egbe et al^11^ in 2018 with 33 patients which was extended to 54 patients from case reports,^25^ all of whom were included in a systematic review totaling 65 patients with biopsy-proven HCC.^26^ Our cohort had a similar age and time between Fontan palliation and HCC diagnosis. The more advanced cancer at diagnosis in the Egbe et al cohort (metastatic disease in 33% vs 5% in our cohort) likely explains the difference in 1-year survival (53% vs 79%).

Survival among Fontan patients with HCC appears worse than survival in non-Fontan HCC. According to published data on HCC in the non-Fontan population, mean survival is approximately 7 to 8 years in BCLC stage 0.^27^ Estimated cumulative median survival is 59 months for BCLC stage A, 24 months for stage B, 9 months for stage C, and 11 months for stage D^28^ which is considerably higher than survival in this Fontan HCC cohort, likely related to multiple factors specific to the FC that lead to lower survival prospects overall including heart failure, sudden death, arrhythmia, etc.^29^

We surmise that choice of HCC treatment modality may be influenced by underlying cardiac disease, which is exemplified by the high burden of cardiac comorbidity observed in the cases. It is possible that curative surgery may be offered less often to HCC patients with FC, contributing an unmeasured bias to survival outcomes. The surgical risk of hepatectomy or liver transplant in the patient with FC is substantial, and while hepatectomy for HCC in this population has been reported,^30^ liver transplant alone is not routinely performed. CHLT—a therapy that addresses both HCC and the cardiac condition in which FALD arises—may be considered, but is a highly specialized treatment with high morbidity and mortality.^31^ This high risk of CHLT in a complex cardiac patient, in contrast to liver transplant alone performed in a non-Fontan HCC patient, could pose a significant barrier to curative transplantation for the patient with FC. It must be noted that HCC was known in only one-third (3 out of 9) of patients who underwent CHLT suggesting they were referred for non-HCC, likely cardiac, indications complicating an analysis of survival by treatment strategy.

The BCLC staging system for liver cancer is linked to prognosis and predictive of survival.^22^ In a first-ever attempt to apply this stratification system to this patient population, we found survival differences by BCLC stage favoring very early and early stage disease, similar to other HCC populations. Though we were unable to identify baseline differences in demographic characteristics or cardiac comorbidities to account for potential confounding, we present an age-adjusted survival analysis given limitations of the sample size. Further research and larger numbers are required to understand contribution of cardiac comorbidities to survival and validate stratification by BCLC stage in HCC patients with FC.

On exploratory analysis, there was no clear survival advantage for those who were under active imaging surveillance. There was, however, an over 4-fold higher odds of being treated with curative intent which could reflect earlier stage at which disease was detected in this subgroup. While the optimal surveillance strategy in patients with FALD has not been determined, we observed that AFP alone would be inadequate. Though elevated AFP was shown to be a predictor of FALD-related HCC in a single-center cohort study,^10^ it is notable that 29% of patients in our cohort had nonelevated AFP with levels <10 ng/dL. Our institutional practice has been, when possible, to obtain MRI with hepatobiliary contrast agent as a baseline study to identify any concerning lesions, and to subsequently follow patients without concerning findings with ultrasound and serum AFP every 6 months. We believe that the creation and adoption of universally agreed-upon protocols for screening are vitally needed.^2^^,^^32^

### Study Limitations

Although this study represents the largest cohort of HCC in those with FC to date, the numbers studied were small and follow-up time relatively short. Therefore, the stratified survival analyses warrant cautious interpretation, especially beyond 1 year, and should be considered exploratory in nature and hypothesis-generating. The crude survival estimates do not account for confounding variables, though in a secondary analysis (not shown), there were no differences in baseline characteristics between groups. There are many other possible factors that could affect such time-to-event analyses including lead- and length-time biases, competing risks of death from other causes, in addition to unmeasured confounding variables given what little is known about the disease.

We acknowledge that case inclusion criteria are not standard. Although HCC can be diagnosed in patients with, or at risk for, cirrhosis by cross-sectional imaging alone,^22^ LI-RADS criteria stipulate that this schema is not applicable to congestive hepatopathy.^12^ FALD is associated with the formation of benign nodules that may resemble HCC,^33^^,^^34^ leading to diagnostic uncertainty and possible false positives. To improve diagnostic accuracy, patients in our study with radiographic LI-RADS 5 lesions, normally categorized as “definite HCC,” required additional parameters, namely elevated AFP or evidence of relatively rapid growth, to be included. We acknowledge that these nonhistologic HCC diagnostic criteria are not validated yet are reflective of a clinical threshold to treat HCC in cases where pretreatment biopsy is not accessible. We performed a sensitivity analysis excluding cases diagnosed by imaging and observed no significant difference in survival between cases diagnosed with or without histologic confirmation.

Last, there is variation in FALD surveillance across centers, and it is conceivable that, in the absence of guidelines, patients deemed “healthy” would not have routine bloodwork or liver imaging performed, representing a potential selection bias for controls and an underestimate of true disease.

## Conclusions

Our study presents a comprehensive analysis of diagnosis, treatment, and outcomes in the largest Fontan patient cohort with HCC to date. Individuals with FC diagnosed with HCC exhibit distinctive features, including significant cardiac comorbidities, advanced FALD with a high prevalence of cirrhotic morphology, all at a relatively young age at diagnosis. Those diagnosed at earlier stages of disease appear to have better survival as well as those who underwent treatment with curative intent. Being under active surveillance showed a nonsignificant trend toward higher survival. It is possible that routine screening could lead to early detection so that curative strategies may safely be pursued. As such, we emphatically urge cross-collaboration between congenital cardiologists, hepatologists, and radiologists to develop and advance a universal HCC screening protocol for the patient with FC.

Perspectives **COMPETENCY IN MEDICAL KNOWLEDGE:** Patients born with single ventricle congenital heart disease who have undergone surgical palliation resulting in FC are at risk for developing HCC. Those with HCC have considerable burden of cardiac comorbidities and advanced FALD. Patients diagnosed with early stage HCC appear to have better survival compared to those diagnosed at later stages.

**COMPETENCY IN PATIENT CARE:** Abdominal imaging by ultrasound, MRI, or CT along with AFP levels can be used to screen for HCC.

**TRANSLATIONAL OUTLOOK:** Further research in the epidemiology and natural history of HCC in the Fontan patient are necessary for the development of universal screening protocols.

## Funding support and author disclosures

This study was supported by a grant from the Children's Hospital of Philadelphia Cardiac Center. Dr Saraf has received funding from 10.13039/100000002NIH K08 HL 161440 and 10.13039/100000968AHA CDA 852875. The authors have reported that they have no relationships relevant to the contents of this paper to disclose.
